# On the transparency of large AI models

**DOI:** 10.1016/j.patter.2023.100797

**Published:** 2023-07-14

**Authors:** Wanying Wang, Ge Wang, Vukosi Marivate, Andrew L. Hufton

**Affiliations:** Scientific Editor, *Patterns*; Department of Biomedical Engineering, Rensselaer Polytechnic Institute; Department of Computer Science, University of Pretoria; Editor-in-Chief, *Patterns*

## Abstract

Scientists using or developing large AI models face special challenges when trying to publish their work in an open and reproducible manner. In this editorial, our journal shares some tips to help researchers in this field understand our current policies and prepare submissions that are as transparent as possible.

As large AI models demonstrate increasingly human-like performance on complex tasks, many scientists are developing or adapting these models to empower their research and applications. Because of the substantial costs involved in building, training, and running large AI models, closed-source models can often offer performance that cannot be matched by open-source counterparts, making them tempting tools for researchers even if they are not transparent or accessible according to conventional academic standards. Moreover, even researchers who are developing their own AI models may face special challenges when trying to publish their work in an open and reproducible manner. In particular, the very large datasets required to train AI models often come with special challenges that make them inherently hard to share—ranging from sheer size to tricky copyright and privacy issues. In this editorial, we share some insights and tips that we hope will help researchers in this field understand our journal’s policies and prepare submissions for the journal.

Whenever possible, we encourage authors who are developing or fine-tuning their own large AI models to openly share the training datasets, final models, and any custom source code used in development. If the full dataset or model cannot be shared, a strong justification needs to be provided and approved by our editors. This is in keeping with our firm commitment to publishing open and FAIR science, and the high peer-review standards of the Cell Press journals, which require that reviewers have the fullest possible access to the authors’ methods and any relevant custom source code. At the same time, authors should understand the complexities and tradeoffs associated with different release choices, as these have an impact on how models can be audited, biases can be identified, and potential harms can be mitigated (see Solaiman, FAccT '23 Proceedings, 111–122).

We recognize that these high standards may pose challenges for researchers who are using commercial, closed-source AI models including the popular models developed by OpenAI. We strongly encourage researchers who are considering using closed models to take into account how this might impact the transparency and reproducibility of their work. Wherever possible, we encourage such researchers to replicate key parts of their study using open-source models, while also taking into account the limitations of such models (see Gudibande et al., arXiv, 2305.15717). We advise researchers developing novel AI models or methods in commercial partnerships to speak early with their partners about the transparency requirements of scholarly publishing and to develop mechanisms that allow models, code, and data to be shared with reviewers and other researchers. The journal reserves the right to decline papers on tools, methods, or other advances that rely solely or mainly on closed-source AI models.

Nonetheless, given the transformative impact that commercial AI models are having on many aspects of our society, we recognize that we cannot ban them from our pages, nor do we wish to. For studies that use these methods to explore topics of particular importance, we will work with authors to find reasonable compromise solutions. As an example, this issue of *Patterns* includes a short piece demonstrating that publicly available tools that purport to detect AI-generated content show clear biases against non-native English speakers (Liang et al., 100779; see also the related preview by Otterbacher, 100796).

For authors who are training or fine-tuning AI models using sensitive datasets with privacy or ethical restrictions—for example, electronic health record data or other clinical datasets—we encourage them to look for ways to provide access to other researchers through a suitable and ethically appropriate controlled-access mechanism. Authors should describe in detail in their papers how other researchers may request access to these kinds of sensitive data, who will consider access requests, and what restrictions may apply. Whenever possible, we encourage authors to host sensitive datasets in formal, controlled-access data repositories that can guarantee preservation and help manage ethical access and use. Researchers working with such data types should always confer with their local institutional ethical guidance and ensure that they comply with all relevant laws and guidelines.

We further encourage our authors to explore other more innovative sharing solutions as a complement or alternative to traditional controlled-access sharing mechanisms. Federated learning and other model-sharing strategies can be used to facilitate collaboration and replication without disseminating sensitive data (see Li et al., Patterns 3, 100603). Simulated data can be used as a surrogate so that readers can see the training and test processes in action, even if raw data cannot be shared (see Jalko et al., Patterns 2, 100271 and Khorchani et al., Patterns 3, 100453). In the future, digital twins or metaverse environments that model, for example, data capture and processing within a hospital environment may offer even more advanced solutions to these kinds of challenges (Wang et al., Nat. Mach. Intell. 4, 922–929).

Authors should be transparent about restrictions, biases, or ethical issues associated with their training datasets and final models. To help with reporting such information, we encourage authors to refer to the information included in “Model Cards,” a transparency concept now in use by many AI model developers (Mitchell et al., FAT∗ '19 Proceedings, 220–229), as well as in “Data Statements,” a similar approach for the source datasets (Bender and Friedman, Trans. Assoc. Comput. Linguist. 6, 587–604). For a comprehensive discussion of bias and unequal performance issues in medical machine learning, along with suggestions for countering such issues, we also refer readers to the perspective by Petersen et al. in this issue (Petersen et al., 100790).

In brief, this editorial represents suggestions and guidelines, not hard rules. The journal will continue to prioritize for peer-review works that we feel are exceptional in terms of their openness and FAIRness. At the same time, we commit to working with our authors to find suitable compromises and to reward creative, out-of-the-box thinking that promotes collaborative, reproducible, ethical, and equitable scientific research.

